# Transcriptome-Wide Analysis Reveals a Role for Extracellular Matrix and Integrin Receptor Genes in Otic Neurosensory Differentiation from Human iPSCs

**DOI:** 10.3390/ijms221910849

**Published:** 2021-10-07

**Authors:** Lejo Johnson Chacko, Hanae Lahlou, Claudia Steinacher, Said Assou, Yassine Messat, József Dudás, Albert Edge, Berta Crespo, Moira Crosier, Consolato Sergi, Anneliese Schrott-Fischer, Azel Zine

**Affiliations:** 1 Inner Ear Laboratory, Department of Otorhinolaryngology, Medical University Innsbruck, Anichstrasse 35, 6020 Innsbruck, Austria; lejo.johnson@i-med.ac.at (L.J.C.); claudia.steinacher@i-med.ac.at (C.S.); jozsef.dudas@i-med.ac.at (J.D.); 2 Harvard Medical School, Massachusetts Eye and Ear Infirmary, Boston, MA 02101, USA; hanae_lahlou@meei.harvard.edu (H.L.); Albert_Edge@meei.harvard.edu (A.E.); 3 IRMB, Univ Montpellier, INSERM, CHU Montpellier, 34000 Montpellier, France; said.assou@inserm.fr; 4 LBN, Laboratory of Bioengineering and Nanoscience, Univ Montpellier, 34090 Montpellier, France; messat.yacine@gmail.com; 5 UCL Great Ormond Street Institute of Child Health, University College London, London WC1 N1EH, UK; berta.crespo@ucl.ac.uk; 6 Human Development Biology Resource, Biosciences Institute, Faculty of Medical Sciences, Newcastle University, Newcastle NE1 4EP, UK; moira.crosier@newcastle.ac.uk; 7 Anatomic Pathology Division, Children’s Hospital of Eastern Ontario, University of Ottawa, Ottawa, ON K1H 8L1, Canada; csergi@cheo.on.ca

**Keywords:** human induced pluripotent stem cells, otic sensory progenitors, gene expression analysis, extracellular matrix, integrins, human fetal inner ear

## Abstract

We analyzed transcriptomic data from otic sensory cells differentiated from human induced pluripotent stem cells (hiPSCs) by a previously described method to gain new insights into the early human otic neurosensory lineage. We identified genes and biological networks not previously described to occur in the human otic sensory developmental cell lineage. These analyses identified and ranked genes known to be part of the otic sensory lineage program (SIX1, EYA1, GATA3, etc.), in addition to a number of novel genes encoding extracellular matrix (ECM) (COL3A1, COL5A2, DCN, etc.) and integrin (ITG) receptors (ITGAV, ITGA4, ITGA) for ECM molecules. The results were confirmed by quantitative PCR analysis of a comprehensive panel of genes differentially expressed during the time course of hiPSC differentiation in vitro. Immunocytochemistry validated results for select otic and ECM/ITG gene markers in the in vivo human fetal inner ear. Our screen shows ECM and ITG gene expression changes coincident with hiPSC differentiation towards human otic neurosensory cells. Our findings suggest a critical role of ECM-ITG interactions with otic neurosensory lineage genes in early neurosensory development and cell fate determination in the human fetal inner ear.

## 1. Introduction

The inner ear sensory organs contain highly specialized mechanosensory epithelia, essential for hearing and balance. The five vestibular organs and the auditory organ, the organ of Corti, originate from the otic vesicle during embryonic development and include various types of sensory hair cells (HCs) and supporting cells. Nerve fibers from the spiral ganglion (SGNs) or vestibular ganglion neurons (both derived from the otic vesicle) innervate auditory and vestibular HCs.

Loss of auditory and vestibular HCs and their associated neurons leads to hearing and balance deficits [1], and these disorders tend to be permanent. There is regeneration of the inner ear neurosensory structures following noise trauma, ototoxic medications, genetic factors or aging [2]. A potential treatment option for these chronic disorders would be the transplantation of in vitro differentiated otic neurosensory progenitors into the damaged inner ear. An alternative would comprise activation of a potential regenerative capacity of otic progenitors to replace lost cells. Establishing chemically defined 2D and 3D culture systems for stepwise in vitro differentiation of human pluripotent stem cells was significant. This progress successfully turned otic neurosensory progenitors [3,4,5] into either HC or SGN-like cells [6,7,8,9], thus recapitulating some aspects of in vivo ear inner ear development. Transplantation of human otic sensory progenitors derived from induced pluripotent stem cells (hiPSCs) has been attempted in the mammalian cochlea, and some engraftment of cells into the sensory epithelium has been reported [10,11]. However, functional integration remains a long-term goal. A lack of data about early otic neurosensory development in the auditory and vestibular sensory epithelia of humans hampers cell therapy development. This study aims to contribute to filling this gap. This study aimed to identify upregulated individual genes and networks in the human otic sensory lineage by high-throughput transcriptomic RNA sequencing (HTS) analysis of hiPSCs derived otic neurosensory progenitors in vitro followed by their in vivo validation in the human fetal inner ear. We found several expressed genes with signatures of extracellular matrix (ECM) and integrins (ITG) during the time course of hiPSC in vitro differentiation toward otic neurosensory fate. In addition, other known inner ear neurosensory genes [i.e., SOX (SRY-related HMG-box), CHD7 (Chromodomain helicase DNA-binding protein 7) and retinoic acid (RA)-related genes] were upregulated in parallel with ECM/ITG genes in differentiated cell cultures. Amongst the key upregulated genes during otic sensory differentiation from hiPSCs, we focused our analyses on a subset of ECM [COL2A1 (Collagen, Type II, Alpha-1), COL9A2 (Collagen, Type IX, Alpha-2), ITGAV (Integrin, Alpha-V)], transcription factors [DACH1 (Dachshund family transcription factor 1), HMGA2 (High Mobility Group AT-Hook 2) and CHD7. These gene families showed cell-type specific developmental expression in human fetal equivalents of the human inner ear. Our findings suggest the importance of the regulation of ECM-ITG gene networks in promoting early otic neurosensory lineage. This work also highlights the usefulness of in vitro study of hiPSC differentiation into otic neurosensory progenitors and in vivo validation of functional genes and active pathways in the human fetal inner ear.

## 2. Results

We have recently described an in vitro culture protocol that generates an enriched otic neurosensory progenitor cell population from differentiation of hiPSCs by stepwise modulation of FGF, TGFβ and WNT signaling pathways [4]. Here, we perform HTS, qPCR and bioinformatic analysis to assess gene expression during human otic sensory differentiation. We uncover known and unknown pathways of cell signaling in otic progenitors and we validate our results by immunohistochemistry in the human fetal inner ear.

### 2.1. Transcriptome Analysis during hiPSC Differentiation into Otic Neurosensory Lineage

**Figure 1:**
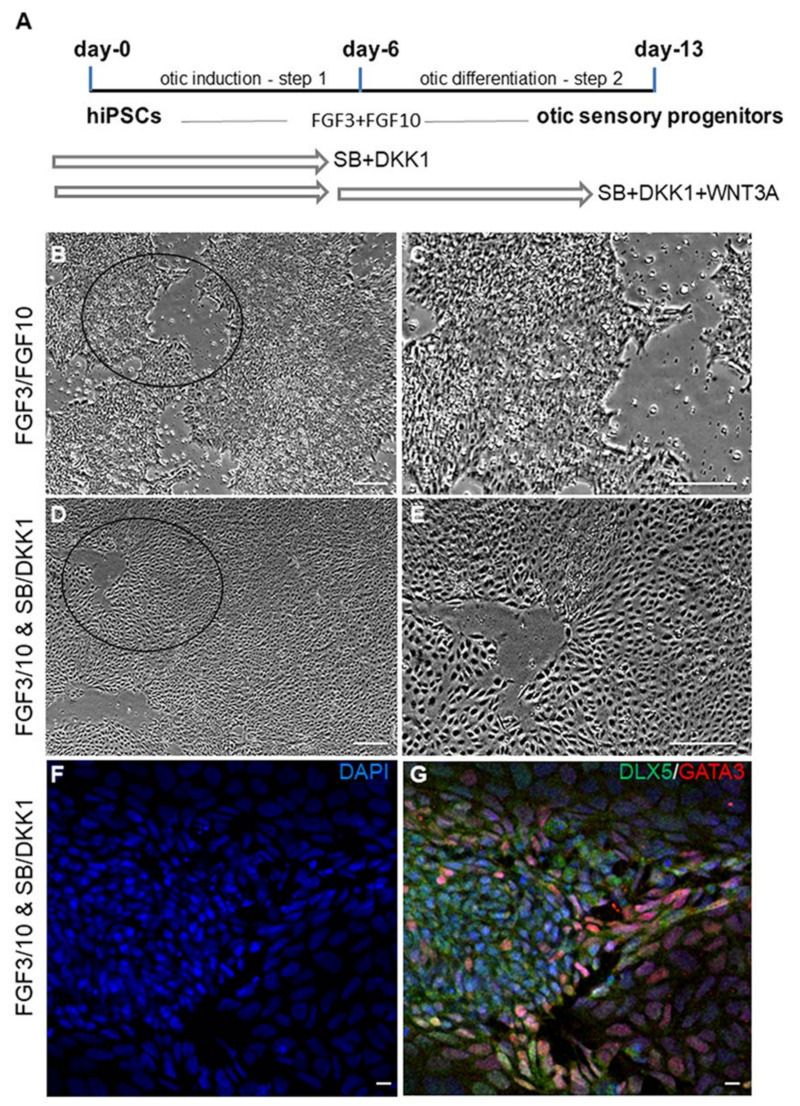
Schematic representation outlining the timeline and culture conditions for otic sensory differentiation from hiPSCs. (**A**) In a first step, undifferentiated hiPSCs were exposed to FGF3/FGF10 growth factors and SB for early otic/placodal induction until day 6 in vitro and, in a second step, they were differentiated into otic sensory cells by exposure to FGF3/10, DKK1/SB and WNT3A until day 13 in vitro. (**B**,**C**) Phase-contrast representative images showing the morphological characteristics of hiPSC-derived otic progenitors exposed to FGF3/10. (**C**) A magnification from the area indicated by a circle in (**B**). (**D**,**E**) Phase-contrast images showing the morphological characteristics of hiPSC-derived otic progenitors exposed to FGF3/10 + DKK1/SB. (**E**) A magnification from the area indicated by a circle in (**D**) showing that partially differentiated hiPSCs at day 6 displayed a homogeneous morphological appearance after exposition to FGF3/10 and SB/DKK1 treatments when compared to the cell cultures exposed only to FGF3/10 (**B**,**C**). (**F**,**G**) Representative immunostainings for the early otic markers DLX5 and GATA3 in day 6 FGF3/10/SB/DKK1-treated cultures. The immunostaining of GATA3 (shown in red) and DLX5 (shown in green) is detected in a subset of differentiated otic/placodal cells. DAPI staining is shown in blue. Scale bars = 200 µm (**B**–**E**); 20 µm (**F**,**G**). Abbreviations: FGF: fibroblast growth factor; SB-431542: TGFβ pathway inhibitor; Dkk1: Dickkopf-related protein-1: WNT pathway inhibitor. WNT3A: Recombinant human WNT ligand.

Undifferentiated hiPSCs in monolayer cultures were treated with SB431542 (TGFβ inhibitor) and Dkk1 (WNT inhibitor) until day 6 in vitro. They were then challenged with WNT3A for an additional 7 days (Figure 1A). Genes associated with pluripotency were progressively repressed, consistent with our previous data showing a marked difference between undifferentiated hiPSCs and the transcriptome of otic sensory cells at day 13 in vitro ([4]; (ENA) database (E-MTAB-6679)). The hiPSCs treated cultures underwent rapid and profound morphological changes leading to homogeneous colonies suggesting their progressive differentiation (Figure 1B–E). The differentiated cells in treated-cultures were analyzed by immunocytochemistry for specific cell type markers along the human otic lineage specification [3,4,8,12,13]. We observed a subset of differentiated cells that expressed *GATA3* and *DLX5* in distinctive and overlapping expression patterns indicating their potential commitment towards otic placodal lineage (Figure 1F,G) as early as day 6 in vitro in SB/DKK1-treated cultures.

**Figure 2:**
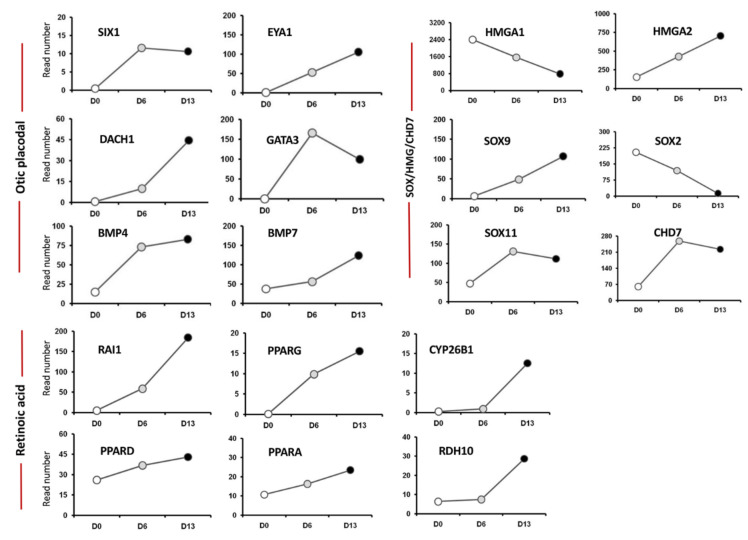
Analysis of cell lineage trajectories by HTS of hiPSCs-derived otic sensory cells. The figure shows read numbers of otic/placodal lineage-specific (*SIX1*, *EYA1*, *DACH1*, etc.), *SOX*/*HMG*/*CHD7* (*SOX9*, *SOX11*, *HMGA2* and *CHD7*) and RA signaling pathway (RAI1, CYP26B1, RDH10,…) gene markers that are gradually enriched during the time course of otic induction at day 6 and day 13 in vitro as compared to undifferentiated hiPSCs at day 0 in vitro.

The HTS analysis of hiPSCs and differentiated cells showed that day 6 and day 13 in vitro signatures were characterized by a progressive increase in the expression of specific gene lineage markers, such as *EYA1*/*SIX1*, *DACH1*, *BMP4*/*7*, key transcription factors in otic placodal fate determination. Members of the *EYA1*-*SIX1*-*DACH1* gene pathway, including *GATA3* and *BMP4*/*7*, are expressed in the ventral, ventromedial, or medial region at the otic pit stage together to specify the otic placodal region [14]. For these genes, differentiated cells showed a significant fold increase in day 6 and day 13 cell culture samples (Figure 2). In addition, the HTS analysis of undifferentiated hiPSCs and differentiated cells showed a subset of upregulated transcription factors, including members of the SOX, HMG, CHD gene families involved in neurosensory development of the inner ear (Figure 2). SOX9 and SOX11 are among crucial otic/placodal gene markers [15,16] upregulated in differentiated cells at day 6 and day 13 cultures, respectively. HMGA2, a member of the HMG family of architectural transcription regulators, facilitating enhanceosome formation on various of mammalian promoters [17], displayed a progressive increase in its expression pattern as hiPSCs differentiated toward otic neurosensory fate. Chromatin remodeling protein, CHD*7*, is one of several proteins essential for proper formation of the mammalian inner ear and brain [18], increased over the time course of hiPSC differentiation (Figure 2). Previous studies reported a potential link between CHD7 and RA signaling in the inner ear, as modulation of RA signaling can attenuate inner ear and neural stem cell defects that arise from CHD7 deficiency [18]. Furthermore, HTS analysis revealed a parallel increase in CHD7 expression and several components (i.e., *RDH10*, *CYP26B*, *RAI1*) of RA signaling in differentiated cells at day 13 in vitro (Figure 2).

The expression of some of these RA signaling genes has been reported in the otocyst and RA may regulate several genes involved in the mesenchymal-epithelial interactions controlling inner ear morphogenesis [19]. In addition, a previous study identified RA and SHH (Sonic hedgehog) as sensory competence factors for bone marrow-derived pluripotent stem cells [20].

**Figure 3:**
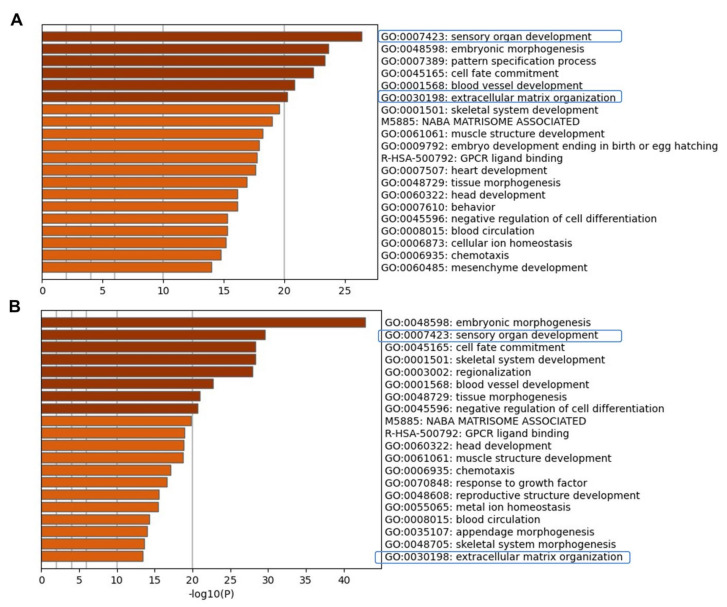
Visualizations of functional enrichment. Metascape bar graph for viewing top enriched ontolog clusters in the lists of up-regulated genes at day 6 (**A**) and at day 13 in vitro (**B**) relative to undifferentiated hiPSCs. Metascape enrichment analysis pinpointed the top most significantly pathways that include sensory organ development, embryonic morphogenesis, pattern specification, and ECM organization as highly enriched in human otic/placodal cells at day 6 and otic sensory cells at day 13 differentiated cell cultures.

To examine active pathways during the time course of hiPSC differentiation in vitro, we performed a gene ontology (GO) analysis. The biological process GO analysis revealed an enrichment of genes related to ECM organization in addition to those of sensory organ development, embryonic morphogenesis, and pattern specification processes (Figure 3 and Figure S1) all of which could play a role in hiPSC differentiation process toward human otic sensory cells.

**Figure 4:**
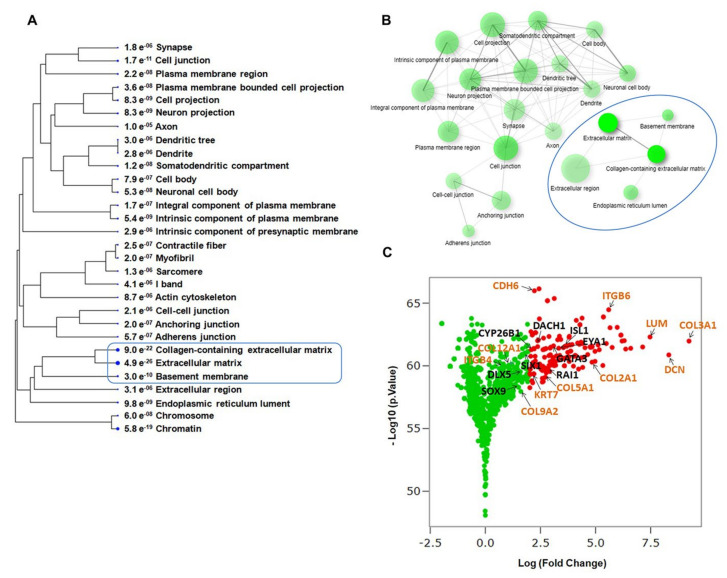
Biological characteristic analysis of top cellular component during hiPSC differentiation into otic sensory cells. (**A**) Cellular component categories of upregulated genes that were significantly enriched in differentiated hiPSCs at day 13 in vitro relative to undifferentiated hiPSCs (*p*-values for the lack of enrichment are shown). The ECM and Collagen-containing ECM are among the top-clustered cellular components. (**B**) Network visualization of enriched cellular components performed by GO analysis revealed the ECM and Collagen-containing ECM are the highly enriched cellular components in the gene signature of otic sensory cells at day 13 in vitro. (**C**) Volcano plots representing the up-regulated genes in differentiated hiPSCs at day 13 in vitro compared to undifferentiated hiPSCs at day 0 in vitro. Adjusted *p*-value (or FDR) Threshold < 0.05 and log2 (Fold Change) >2. Labeled are differentially upregulated genes with log2 (FC)>2 (red dots) that includes ECM and ITG gene members.

The cellular component GO analysis also identified ECM, collagen-containing ECM, and basement membrane among highly-enriched cellular components at day 13 in vitro (Figure 4A,B), suggesting that components of these non-otic pathways could be activated and contribute in cooperation with otic developmental genes to the conversion of hiPSCs into otic sensory lineage.

As an additional criterion of biological relevance, we assessed gene expression changes in differentiated hiPSCs at day 13 in vitro compared to undifferentiated hiPSCs at day 0 in vitro. The volcano plots (Figure 4C) provide a clear view of the genes that were upregulated in day 13 Vs undifferentiated hiPSCs. Interestingly, in addition to otic placodal (i.e., GATA3 and EYA1) genes, ECM (i.e., COL2A1, DCN, COL3A) and ITG such as ITGB6 were upregulated in differentiated cells at day 13 cultures

**Figure 5:**
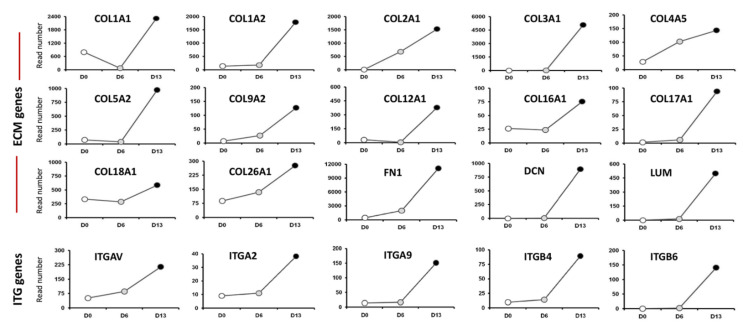
Representative HTS expression profiles for extracellular martix (ECM) and integrins (ITG) gene family members that are progressively enriched in day 6 and day 13 differentiated cell cultures. The HTS revealed an upregulation of a panel of known (*COL1A1*, *COL1A2*, *COL2A1*, *COL9A2*) and unknown (*COL3A1*, *COL5A2*, *COL12A1*, etc.) ECM genes. The HTS analysis also showed upregulation of a subset of known (*ITGAV*, *ITGB4*) and unknown (*ITGA2*, *ITGA9*, etc.) ITG genes in the mammalian embryonic inner ear.

Altogether, our HTS analysis highlighted a panel of differentially expressed genes of the ECM and ITG gene families at day 6 and day 13 cell cultures (Figure 5). We identified the expression of several Collagen-encoding genes that have been previously reported in the inner ear (*COL1A1*, *COL1A2*, *COL2A1*). The second group of unknown ECM genes included a subset of additional variants of collagens (*COL3A1*, *COL5A2*). Other upregulated genes during in vitro differentiation of hiPSCs comprised *DCN* and *FN1* genes, two pivotal ECM effectors [21]. It is interesting to note that the HTS screen also revealed several upregulated ITG gene targets (Figure 5); some of them are known to be expressed in the mouse developing inner ear [22,23] such as the integrin b4 (*ITGB4*), b6 (*ITGB6*), and av (*ITGAV*) subunits. Unknown ITG genes in the inner ear included *ITGA2* and *ITGA9*. The concomitant upregulation in the expression of ECM and their ITG receptors across the time course of hiPSC differentiation implicates these genes in promoting human otic sensory lineage.

### 2.2. Validation of HTS Results by Quantitative PCR

**Figure 6:**
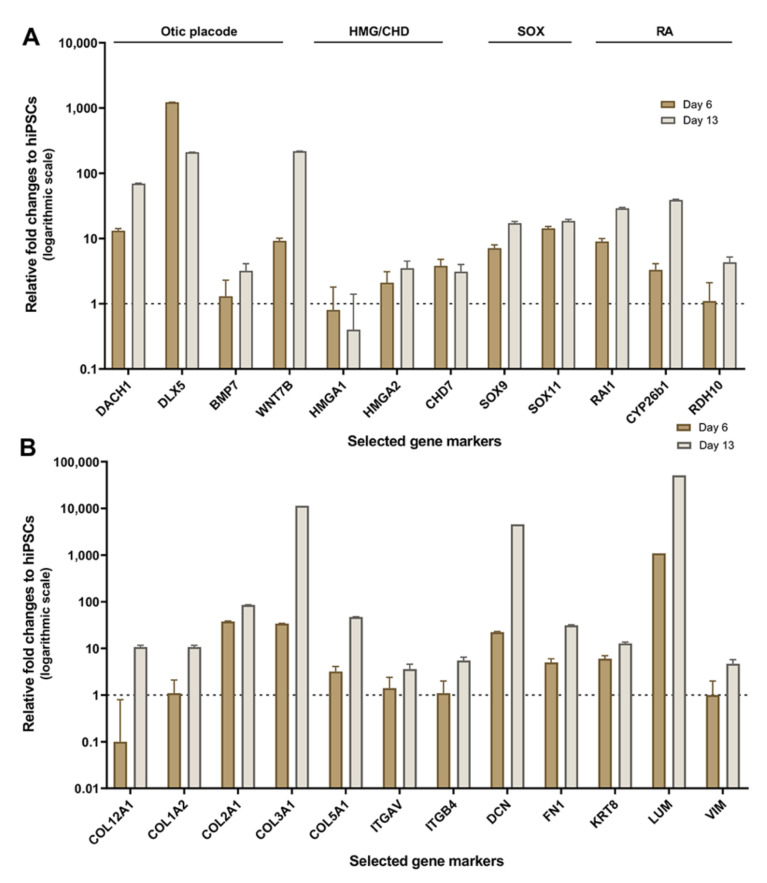
Validation of HTS results by real-time qRT-PCR. (**A**) qPCR expression profiles in day 6 to day 13 culture samples relative to undifferentiated hiPSCs (day 0) were plotted as fold change. Bar charts represent the relative gene expression levels obtained for distinct panels of markers featuring members of otic/placodal, HMG/CHD7, SOX and RA gene families, respectively. Noticeably, results confirm the upregulation of otic/placodal (*DACH1*, *BMP7*, etc.) HMG (*HMGA1, HMGA2,* etc.) CHD7, SOX (*SOX9*, *SOX11*, etc.) and RA (*RAI1*, *RDH10*, etc.) genes in differentiated cells at day 6 and day 13 in vitro, which is the condition that yielded optimal otic sensory induction. (**B**) qPCR expression profiles in day 6 to day 13 culture samples showing a panel of upregulated ECM (*COL1A*2, *COL2A1*, *COL3A*1, etc.) and ITG (*ITGAV*, *IGTB4*) genes during the time course of hiPSCs differentiation in vitro confirming HTS results. Relative gene expressions were calculated according to the 2^−(ΔΔCt)^ method normalized with each gene expression at day 0 in vitro. Data are represented in logarithmic scale with the error bars represent SD of five technical replicates.

Gene panels for this analysis were selected from HTS transcriptomic data on the basis of their potential otic morphogenesis role and the lack of information on the genes in previous studies of the human inner ear. To validate the differences in expression among hiPSC differentiation stages revealed by HTS analysis, we used quantitative PCR (qPCR) to examine the expression of selected potent otic and ECM/ITG gene markers that displayed differential expression patterns (Figure 6A,B). Our qPCR analysis on cell culture samples at day 0, day 6 and day 13 in vitro showed differential expression and good agreement with HTS transcriptomic results. Similar to HTS, the qPCR analysis confirmed a relative increase in the expression of representative gene members of ECM (*COL1A2*, *COL2A1*), ITG (*ITGAV*, *ITGB4*) and transcription factors (*DACH1, SOX9, HMGA2*) during hiPSC differentiation. In addition, members of the RA (*RDH10*, *CYP26b1*) gene family known to be expressed in the otic placode and vesicle [19] showed a relative increase in their expression at day 6 and day 13 differentiated cultures.

### 2.3. Evaluating Relationships among Differentially Expressed Genes through Network Building

Ingenuity pathway analysis (IPA) software was used for the functional assessment of genes that were differentially expressed in differentiated cells at day 13 compared to those of undifferentiated hiPSCs at day 0. Enriched gene ontology (GO) terms revealed that some functional annotations were more represented in cellular and tissue development, cellular movement, and cell-to-cell signaling pathway categories (Figure S2).

**Figure 7:**
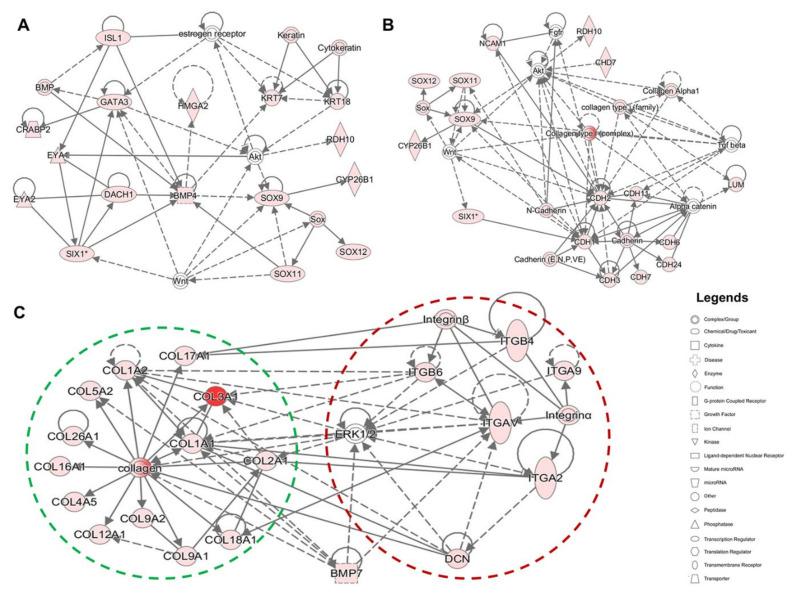
Functional network analysis of upregulated genes during the time course of human otic sensory differentiation in vitro. (**A**,**B**) The most significant IPA network assembled around otic/placodal gene (*EYA1*, *SIX1*, *DACH1,* etc.) markers and their direct and indirect interactions with SOX (*SOX9*, *SOX11*), RA (*CYP26B1*, *RDH10*) and *HMGA2* genes in day 13 differentiated cell cultures. (**C**) The most significant IPA network assembled around ECM and their cellular integrin receptors (ITG) in day 13 cultures showing the networks of *COL1A1* and *COL2A1* and their close interactions with other Collagen and DCN genes. Furthermore, the network showed additional direct and indirect interactions between ECM and ITG genes. *ERK1/2* and *BMP7* genes displayed both ECM and ITG indirect interactions. Nodes shaded in pink represent protein-coding genes that are upregulated in day 13. The intensity of the node color indicates the degree of up-regulation. Edges (lines) and nodes are annotated with labels that illustrate the nature of the relationship between genes and their functions. A solid line represents a direct interaction and a dotted line an indirect interaction.

To explore the functions of genes that were upregulated in day 13 cultures, we identified networks that were constructed around differentially expressed otic placodal (Figure 7A and Figure S3), SOX/CHD7 (Figure 7B and Figure S4), and COL/ITG (Figure 7C and Figure S5) gene transcripts. This analysis revealed that numerous otic/placodal genes were interconnected to members of the ECM and ITG gene families, suggesting their cooperative role during otic sensory differentiation from hiPSCs. The Collagens and ITG genes displayed functional interactions with *BMP7* and *DCN* genes, forming a tightly related network (Figure 7C). It is interesting to note that ECM and ITG were directly or indirectly connected to the otic placodal (*SIX1*, *DACH1*, *BMP4*), RA (*CYP26B1*, *RDH10*) and SOX (*SOX9*, *SOX11*) gene networks. Overall, these results show that ECM/ITG are highly linked to SOX gene networks which play a significant role in neurosensory development and cell fate determination of the mouse and human inner ear [15,16]. Additional upregulated genes, such as *CHD7*, *HMGA*2 and *DACH1* known to be expressed within the mouse embryonic inner ear are also found within networks built around upregulated members of the ECM/ITG gene families (Figure 7A,B). Altogether, the resulting network provides new insights into ECM/ITG interactions during otic sensory differentiation from hiPSCs.

### 2.4. Immunohistochemical Validation of Select Markers in Human Fetal Inner Ear

**Figure 8:**
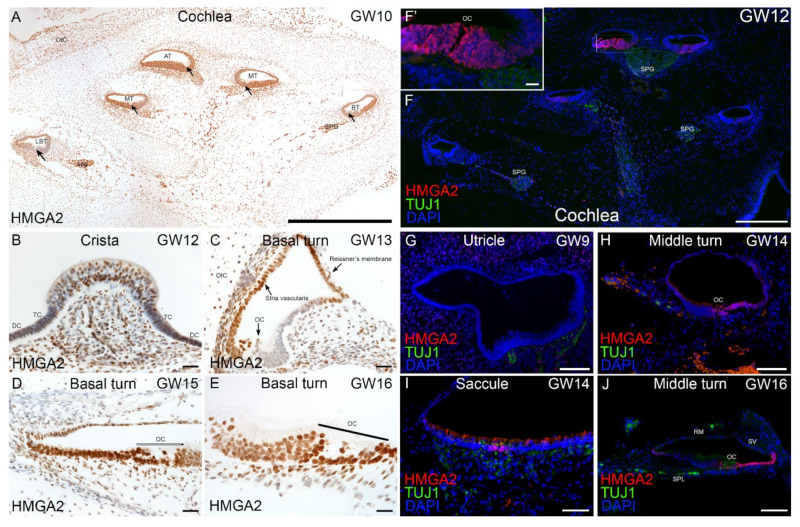
Immunostaining for HMGA2 in human inner ear fetal tissue. (**A**) Overview of immunostained cochlea at GW10. Intense immunostaining for this marker present at middle turn (MT), basal turn (BT) and apical turn (AT) of cochlea as well as in spiral ganglion (SPG). A moderate immunoreaction observed at lower basal turn (LBT). Organ of Corti (arrows). (**B**) Crista ampullaris at GW12. Lowered immunoreactivity with intense expression amongst supporting cells of the crista. Transition cells, TC; dark cells, DC. (**C**) Basal turn of cochlea at GW13. HMGA2 immunostaining is restricted to select regions including future stria vascularis, Reissner’s membrane and nerve fibers. Supporting cells and future organ of Corti (OC) are devoid of immunoreactivity. A few cells of spiral ligament and chondrocytes of otic capsule (OtC) are still immuno+. (**D**) Basal turn of cochlea at GW15. Immunostaining is evident in all supporting cells, the future cells of stria vascularis and HCs of the organ of Corti (OC). (**E**) Basal turn of cochlea at GW16. The HMGA2 staining is clearly visibly in all supporting cells and HCs in the organ of Corti. (**F**) Prominent expression of HMGA2 at GW12 observed in apical portion of cochlea (refer Figure 8F,F’) in region specific to future organ of Corti. Expression pattern of HMGA2 at middle and basal turns of cochlea at this stage appears diminished. Beta III tubulin immunostaining observed in nerve fibers of spiral ganglia (SPG). (**G**) At GW9 absence of HMGA2 immunostaining observed in sensory epithelia of utricle. Beta III tubulin immunoreactivity observed in nerve fibers underlying utricular sensory epithelia with few cells intensely expressing HMGA2 adjacent to these nerve fibers. (**H**) At GW14, increased expression of HMGA2 observed at cochlear middle turn in supporting cells as well in the future spiral prominence. Beta-III tubulin immunoreactivity observed in nerve fibers projecting towards the organ of Corti (OC). (**I**) At GW14, expression of HMGA2 observed in HCs of the saccule as well as in a few supporting cells in a striolar area. Beta-III tubulin immunoreactive fibers are penetrating sensory epithelia of saccule at this stage of development. (**J**) At GW16, expression of HMGA2 is present in supporting cells in a region corresponding to the Claudius cells, Hensen’s cells and reach as far as spiral prominence. Additionally, cells of inner sulcus are immuno+ at this stage. Nerve fibers penetrating organ of Corti (OC) are Beta III tubulin immuno+. Reissner’s membrane (RM), Stria vascularis (SV) Spiral lamina (SPL). Scale bars: 500 μm (**F**); 200 μm (**A**); 100 µm (**E**,**G**–**J**); 20 µm (**B**–**D**,**F’**).

**Figure 9:**
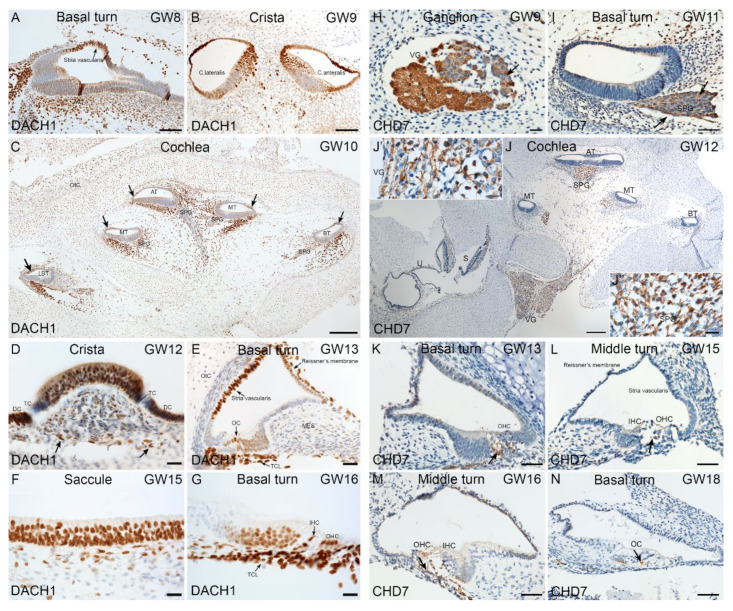
Immunostaining for DACH1 and CHD7 in human inner ear fetal tissue. (**A**) Basal turn of cochlea at GW8. Nuclei of future marginal cells (arrow) in stria vascularis are immuno+ for DACH1. Additionally, mesenchyme cells show strong nuclear staining. (**B**) Cristae ampullaris at GW9. The HCs of lateral and antral crista exhibit strong immunoreactivity in sensory epithelium. Single layered cuboidal epithelial cells of membranous labyrinth are immuno+. Few immuno+ cells observed in the mesenchyme as well. (**C**) Mid-modiolar plane of cochlea at GW10. Strong immunostaining present in mesenchyme underlying sensory epithelium. Few cells of spiral ganglion exhibit immunoreactivity for DACH1. Future stria vascularis (arrows) is immuno+ for this marker. (**D**) Crista ampullaris at GW12. Immunoreaction is present in vestibular HCs and supporting cells. Non-myelinating Schwann cells (arrow) underlying sensory epithelium appear immuno+. Transitional cells (TC) are devoid of immunoreactivity. The dark cells (DC) appear immuno+. (**E**) Cochlear basal turn at GW13. Stria vascularis and Reissner’s membrane immuno+. In organ of Corti (OC), HCs and supporting cells are immuno+. Cells beneath the basilar membrane forming the future tympanic cover layer (TCL) cells are immuno+. (**F**) Sensory epithelia of saccule at GW15. The HCs and supporting cells show a strong immunoreactivity for DACH1. (**G**) Cochlea basal turn at GW16. Outer (OHC), inner hair cells (IHC) and supporting cells are immuno+ for DACH1. Tympanic cover layer (TCL) cells exhibit strong immunoreactivity. (**H**) CHD7 staining in the vestibular ganglion (VG) gestational week (GW) 09. Strong staining in the nerve fibers (arrow). Cytoplasm of the vestibular ganglion cells are also immuno+. Satellite glial cells surrounding the vestibular ganglions are devoid of immunoreactivity. (**I**) Basal turn of cochlea at GW11. Immunoreactivity is present in the nerve fibers (arrows) of the spiral ganglion (SPG) at this stage. (**J**) Overview of the temporal bone at GW12. High immunoreaction observed in the nerve fibers and spiral ganglion at the apical turn (AT) and middle turn (MT). Lowered signal in the basal turn (BT). In the vestibular ganglion, a strong immunostaining is evident at this stage. In the saccule (S) and utricle (U), immunoreactivity is absent. (**J’**) Insert with higher magnification from the vestibular ganglion where the staining of the nerve fibers is evident. (**J’’**) Insert with higher magnification from the spiral ganglion (SPG). Strong immunostaining observed in the nerve fibers. (**K**) Basal turn of the cochlea at GW13. CHD7 are penetrating the organ of Corti (arrow). Outer hair cells (OHC). (**L**) Middle turn of cochlea at GW15. Only single nerve fibers staining observed at the base of the outer hair cells (OHC). Inner hair cell (IHC) (arrow). (**M**) Middle turn of cochlea at GW16. The tunnel of organ of Corti is already open. We can recognize the nerve fibers staining (arrow). (**N**) Basal turn of cochlea at GW18. CHD7 staining is penetrating the IHCs (arrow). Organ of Corti (OC). Scale bars: 200 μm (**C**,**J**); 100 µm (**A**,**B**,**F**,**G**); 50 µm (**I**,**K**–**N**); 20 µm (**D**,**E**,**H**,**J’**,**J’’**).

**Figure 10:**
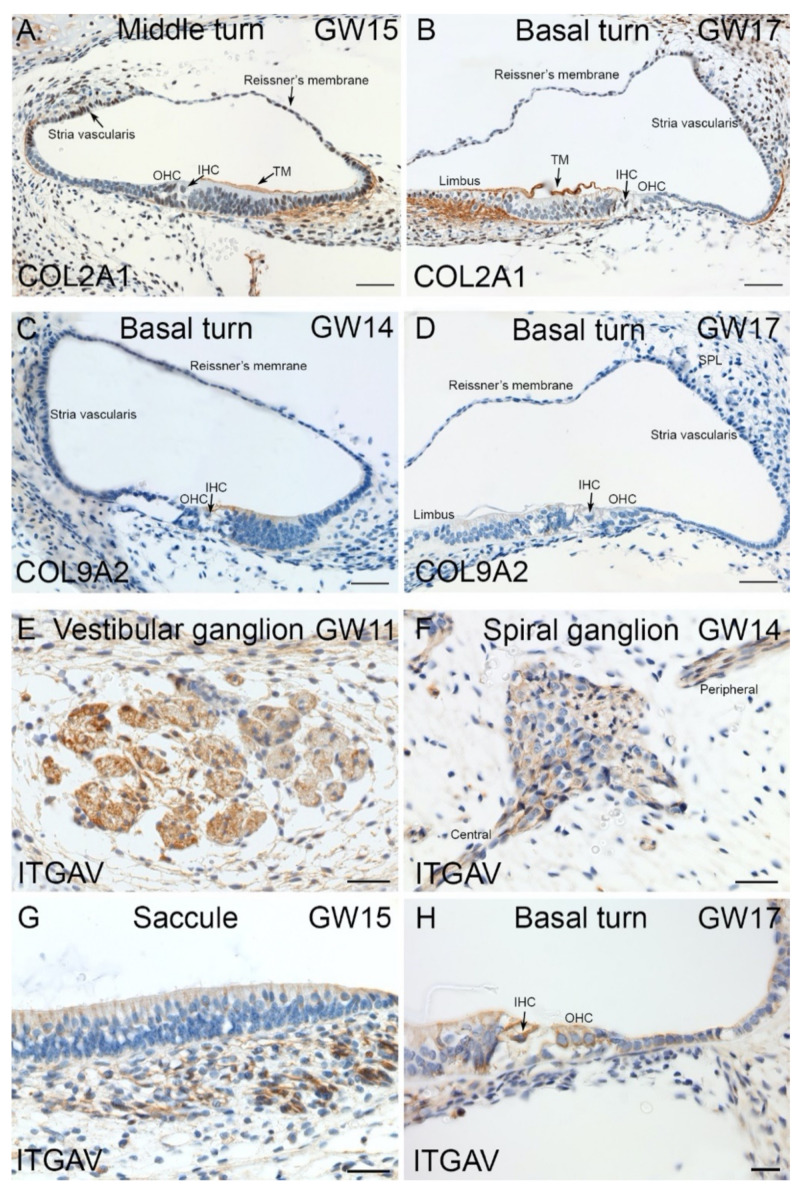
Immunostaining for ITGAV, COL2A and COL9A2 in human inner ear fetal tissue. (**A**) Collagen 2 alpha 1 expression present at GW15 in fibrocytes of the spiral limbus, the future tectorial membrane (TM) also in type I fibrocytes that underlie the stria vascularis region (arrow), Outer (OHC) and inner hair cells (IHC) are devoid of immunoreactivity. (**B**) Basal turn of cochlea at GW17. High expression of Collagen type 2 alpha 1 observed in fibrocytes of spiral limbus and stria vascularis. Tectorial membrane (TM) Inner hair cell (IHC) and Outer hair cells (OHC) are devoid of immunoreactivity. (**C**) Basal turn of cochlea at GW14. Collagen 9-alpha2 immunoreactivity observed for first time in the organ of Corti (OC). Reissner’s membrane (RM), stria vascularis, spiral ligament (spl), Inner (IHC) and Outer hair cells (OHC) labeled. (**D**) Basal turn of cochlea at GW17. COL9A2 expression absent from the organ of Corti (OC). Inner (IHC) and Outer hair cells (OHC) labeled. (**E**) ITGAV immunostaining in the vestibular ganglions at GW11. (**F**) Spiral ganglion of cochlea at GW14. The spiral ganglion neurons and nerve fibers projecting towards central and peripheral nervous systems are immuno+. (**G**) Integrin alpha V expression observed in the nerve fibers underlying sensory epithelium of the developing saccule at GW15. (**H**) Basal turn of cochlea at GW17 with both inner and outer HCs are immuno+ to ITGAV. Both inner (IHC) and outer hair cells (OHC) labeled. Scale bars: 100 µm (**A**–**G**); 50 µm (**H**).

Transcriptomic data collected from otic induction cultures predicted expression and translation of genes occurring throughout the human inner ear during early fetal development. We used immunohistochemistry as a tool to validate this predicted expression and to confirm the translation of a subset of genes in the human fetal inner ear. Selected genes examined include transcription factors like HMGA2, DACH1, CHD7 and, ECM/ITG markers like COL2A1, COL9A2, and ITGAV (Figure 8, Figure 9 and Figure 10). Using chromogenic immunostaining, we investigated the expression of HMGA2 in developing human inner ear from gestational week (GW) 10 to GW16. In vestibular end organs, immunoreactivity present in HCs and supporting cells of cristae ampullaris. At GW10, a gradient of immunoreactivity was observed in a mid-modiolar section of the cochlea with intense expression at apical and middle turns (Figure 8A). At GW12, a defined immunostaining pattern was observed in supporting cells of crista ampullaris and in the mesenchyme (Figure 8B). By GW13, in basal turn of the cochlea, the HMGA2 expression is limited to stria vascularis, Reissner’s membrane as well as a few cells of spiral ligament (Figure 8C). By GW15, the staining is restricted to the supporting cells, the HCs and cells of the stria vascularis (Figure 8D). At GW16 with higher magnification, the immunoreactivity is similar to GW15 with specific staining in the organ of Corti with inner and outer HCs and supporting cell area (Figure 8E). Additionally, using fluorescence imaging at GW12 a more severe gradient in HMGA2 expression was observed in the mid-modiolar plane of the cochlea. HMGA2 immunostaining is most intense at apical turn of cochlea while at cochlear basal turn immunostaining is reduced (Figure 8F,F’). In order to observe nerve fibers at this stage, an antibody specific to Beta III tubulin is also used. At GW9 the utricular sensory epithelia is devoid of HMGA2 expression (Figure 8G). HMGA2 expression at GW14 is limited to future supporting cells and spiral prominence of the cochlea (Figure 8H), while in the saccule, it is restricted to striolar region of sensory epithelia (Figure 8I). By GW16, cochlear supporting cells (Claudius cells, Hensen’s cells) express this protein with immunostaining present at the spiral prominence. Few cells of the inner sulcus were also immunostained for this marker (Figure 8J).

Similarly, we investigated DACH1 expression in the developing human inner ear using chromogenic immunostaining. At the earliest developmental stage examined (GW8), expression of DACH1 in the cochlea is present in future marginal cells of stria vascularis and mesenchyme (Figure 9A). Vestibular HCs are immuno+ for DACH1 in crista ampullaris at GW9. Single layered cuboidal epithelial cells of the membranous labyrinth are also immuno+ for DACH1 (Figure 9B). At GW10, the stria vascularis, the mesenchyme and a few cells of the spiral ganglion are immuno+ for this marker (Figure 9C). Vestibular HCs, supporting cells, dark cells of crista ampullaris are immuno+ at GW12. Few myelinating Schwann cells underlying the crista were also immuno+ (Figure 9D). Between GW13 and GW16, the staining pattern in the cochlea remains similar to previous stages. The exception being the immunoreactivity appearing in cochlear HCs and supporting cells (Figure 9E,G). Vestibular HCs and supporting cells were also immuno+ for DACH1 at GW15 (Figure 9F). The CHD7 expression also examined in the developing human inner ear using chromogenic DAB staining. Scarpa’s ganglion is immuno+ at the earliest week examined (GW9). Nerve fibers and cytoplasm of vestibular ganglia are immuno+ for CHD7. Satellite glial cells lacked immunoreactivity (Figure 9H). At GW11, nerve fibers in spiral ganglia are immuno+ for CHD7 (Figure 9I). In the cochlea, nerve fibers display an immunoreaction gradient for CHD7 with intense reactivity at apical and middle turns by GW12. Sensory epithelia of utricle and saccule were devoid of immunoreactivity (Figure 9J). Vestibular and spiral ganglia are, however, immuno+ for CHD7 (Figure 9J’,J’’). At later gestational weeks examined [GW13 (Figure 9K), GW15 (Figure 9I), GW16 (Figure 9M)] and the cochlea nerve fibers entering the sensory epithelia at the base of IHCs and OHCs.

Additionally consistent with HTS and qPCR data, we found immunoreaction of ECM/ITG markers in human fetal inner ear. Collagen, type II, alpha 1 (COL2A1) expression observed at GW12. The immunostaining observed in fibrocytes that underlie the organ of Corti. At GW13, we detected an expression gradient from the basal (high expression) to the apical (low expression) turn. In the following stages GW14 and GW15, the immunoreaction for COL2A1 is present in the tectorial membrane, mesenchymal cells below basilar membrane and fibrocytes near the stria vascularis (Figure 10A). By GW17, a strong immunoreaction of Collagen type II alpha 1 was detected in fibrocytes of the spiral limbus and stria vascularis (Figure 10B). The COL9A2 is the other collagen type we examined showed first a slight immunoreactivity in the organ of Corti at GW14 (Figure 10C). However, by GW17, this staining for COL9A2 was absent in the organ of Corti (Figure 10D). The integrin alpha V (ITGAV) immunostaining observed mainly among the vestibulum and cochlea’s nerve fibers and ganglia. Strong immunoreactivity for ITGAV is observed in the vestibular ganglions and fiber bundles at GW11 (Figure 10E). The cytoplasm of the cochlear spiral ganglia are immuno+ for ITGAV at GW14. The nerve fibers projecting towards the central and peripheral nervous systems are also immuno+ for ITGAV at this stage (Figure 10F). By GW15, the nerve fibers underlying the saccule are also immuno+ for ITGAV (Figure 10G). The cytoplasm of both IHCs and OHCs are immuno+ for this marker at GW17 (Figure 10H).

Although a limited number of ECM/ITG and transcription factors was tested, immunohistochemistry suggests that gene expression at the mRNA level generally reflects protein expression. Altogether, our data indicate that within the fetal inner ear, expression of ECM/ITG and transcription factors has a markedly higher expression in otic sensory cells and non-sensory otic cells, particularly in domains possibly derived from dorsal and/or ventral otic vesicle regions. Furthermore, the wide dynamic range of ECM/ITG expression in the fetal inner ear makes these genes attractive candidates for regulatory otic neurosensory and other otic lineage studies.

## 3. Discussion

The inner ear develops from the otic placode, a thickened region of cranial ectoderm that invaginates to form the otic cup followed by the otic vesicle [24]. Several signaling pathways, including FGF and WNT have been shown to promote otic placode fate and determinations of neuronal and prosensory otic populations [14]. Thus, systematic HTS of otic lineage distinguishing genes would contribute to our understanding of transcriptional states and gene expression regulations during inner ear development. Furthermore, a large-scale identification of non-otic genes upregulated with known otic lineage markers would greatly benefit studies of inner ear differentiation and regeneration [25].

In this study, HTS analysis showed that evolutionarily conserved transcription factors such as *EYA1*, *DACH1*, *SIX1*, *GATA3* expressed in early otic lineage in vivo [14,24] were upregulated during hiPSC differentiation at day 6 in vitro. This first step of in vitro differentiation is likely to be close to embryonic day E10-11 of mouse embryonic development and gestational week GW5-6 of human fetal development. In contrast, by day 13 in vitro, we showed a progressive upregulation of a panel of non-otic neurosensory lineage markers (i.e., *ECM* and *ITG*) in parallel to upregulation of key otic lineage (i.e., *SOX2/HMG, RA/CHD7*) at day 13 in vitro. This second of step differentiation yielded otic sensory cells likely equivalent to a state of differentiation at E15-16 mouse embryonic development and GW10-11 otic cells from the sensory epithelia of human inner ear [12,26]. We validated the expression of a subset of selected gene markers by qPCR analysis and immunohistochemistry in the in vivo human fetal inner ear.

### 3.1. Enrichment of ECM and ITG during hiPSC Differentiation into Otic Sensory Lineage

The ECM is a multifunctional network of fibrous, gel like-material. Its main components are collagens, glycoproteins and proteoglycans [27]. The ECM provides structural support for organs and tissues, cell layers in the form of basement membranes, and individual cells as substrates. ECM proteins have also been implicated in many cellular processes ranging from cell growth, differentiation, and apoptosis to stem cell fate decisions [27]. Their transmembrane receptors are the integrins that bind to matrix ligands to initiate signals that modify cell adhesion and gene expression, with the responses depending on cell and integrin types [28]. ECM has also been implicated in facilitating morphogen signaling mediated by ligands such as FGF and WNT in controlling movement and presentation of growth factors [27,28]. In comparing the transcriptomes of mouse and human, ECM genes in the CNS, distinct panels of collagens were heavily expressed in dividing neural precursors [29]. Similarly to other organ systems, the mammalian inner ear development involves a complex series of cell-cell and cell-ECM interactions [22]. The mechanical cues from the ECM have been recently reported to promote the survival, expansion and differentiation of inner ear progenitors, partly through activation of WNT signaling [30]. Our HTS screen identified ECM-encoding genes that have been previously reported in the mouse inner ear, such collagen as (*COL1A1*, *COL1A2*, *COL2A1*, *COL9A2*) [31,32]. In addition, the HTS revealed the second group of unknown ECM components in the inner ear that included additional types of collagen (*COL18A, COL26A1*), glycoprotein (*FN1*), and proteoglycan decorin (*DCN*).

In the inner ear, *COL2A1* expression has been detected in both ectodermal and mesodermally derived structures in mammals [33] including connective tissue of neuronal cells, stria vascularis and organ of Corti [34]. The *COL9A2* expression was observed in the otic vesicle at E10.5 and in organ of Corti and spiral ligament during mouse postnatal development [32]. Furthermore, HTS screen also revealed several ITG targets that were enriched in day 13 cultures. The upregulation was most pronounced for *ITGB4*, *ITGAV* and *ITGB6*, suggesting a role for integrin signaling through a complex interaction with ECM ligands during hiPSC differentiation towards otic sensory cells. Of interest, our screen identified new ITG gene candidates, i.e., *ITGA2* and *ITGA9* upregulated during hiPSC differentiation and implicated these ITG genes in morphogenesis of the developing otocyst and inner ear. In the embryonic inner ear, the expression of *ITGB4* was weakly detected at E10.5 in medial region of the mouse otocyst [35]. Another study demonstrated mRNA expression for *ITGAV* in HCs at E16.5 during the mouse inner ear development [23]. Integrin signaling has been involved in the proliferation of neuroepithelial progenitors and neurogenesis [36]. Using oligonucleotide inhibition in the developing cochlear tissue of rats, it was demonstrated that *ITGAV* and *ITGB4* significantly decreased the transcription of nuclear factor-kappa-B, a signal molecule involved in growth and proliferation induced by EGF and bFGF [37]. Previous analysis revealed that ITGB1-expressing cells secrete WNT7A that induces the expression of DCN in the neighboring β1 integrin-negative cells [38]. Furthermore, experiments with explants using inhibitors and genetic knock-downs in vivo revealed an ITG-WNT7A-DCN pathway that promotes proliferation and differentiation of neuroepithelial cells and identifies DCN as a novel neurogenic factor in the CNS [36]. Interestingly, we have previously demonstrated upregulation of several WNT ligands (i.e., WNT5A, WNT5B, WNT7B) during hiPSC differentiation [4]. Altogether, these results suggest a functional link between WNT and *COL/ITGB/DCN* networks during otic neurosensory differentiation from hiPSCs.

### 3.2. Enrichment of CHD7-RA during Otic Sensory Differentiation

The inner ear is particularly sensitive to *CHD7* levels and is the most commonly affected organ in individuals with CHARGE syndrome [18]. Using animal models, it has been shown that modulation of the expression of RA signaling during embryogenesis leads to developmental defects similar to those in CHARGE syndrome, suggesting critical cooperative roles for *RA* and *CHD7* in a common pathway to regulate inner ear development [18]. We observed semicircular hypoplasia/aplasia and shortened cochlear duct characteristic of a Mondini malformation in a temporal bone of a 3 month old child with CHARGE syndrome [39]. In this study, we found that differentiated cells at day 13 in vitro exhibited a relative upregulation of *CHD7* suggesting that the change in *CHD7* expression is potentially involved in the acquisition of early otic neurosensory identity. The *CHD7* binds to thousands of promoter and regulatory regions in the genome and been shown to activate the expression of several downstream targets, including *SOX9*/*SOX11* [40] that were also upregulated during hiPSC differentiation.

### 3.3. Enrichment of SOX/HMGA during Otic Sensory Differentiation

The SOX proteins regulate gene expression, acting as either transcriptional activators or repressors, in multiple tissues, and play crucial roles in various developmental processes [41]. The spatial expression pattern of SOX11 in the fetal brain suggests that SOX11 is involved in neurogenesis since its expression was higher in the ventricular zone than in areas with relatively low levels of neurogenesis [42]. Mutations in human *SOX9* result in skeletal abnormality, sex reversal syndrome, campomelic dysplasia (CD), which has a high lethality rate. Sensorineural hearing loss has been reported in the surviving CD patients, suggesting a role of *SOX9* in inner ear development [43]. In the mouse otocyst, SOX9 and SOX11 expression overlapped with *SOX2* in the prosensory and sensory regions. This initial overlap but subsequent differential expression of *SOX2*, *SOX9* and *SOX1*1 in the sensory epithelia suggested that they may have distinct roles in developmental pathways that direct cells towards different otic fates [44]. Furthermore, it was reported that SOX proteins (i.e., SOX4, SOX11) were expressed throughout the prosensory domains of the otic vesicles and were necessary for proliferation of progenitors and differentiation of HCs in the sensory epithelia of the mouse inner ear [16]. Thus, the upregulation of *SOX9* and *SOX11* and downregulation of *SOX2* during hiPSC differentiation may reflect a more otic sensory transcriptional identity of differentiated cells. We can only speculate about the final fate of these cells, as we were not able to follow individual cells in our in vitro experimental setup. The HMGA are small non-histone proteins that can bind DNA and modify chromatin state [45]. In general, HMGA is expressed in ESCs and some adult stem cells. Numerous experimental data have indicated that they play a fundamental role in regulating differentiation in various of lineages [17]. HMGA1 is expressed in ESCs and its levels decline during differentiation [46]. In contrast, HMGA2 is expressed at high levels in ESCs and is further upregulated during differentiation [47]. Interestingly, in our HTS and qPCR analyses, these genes showed an opposite trend in their relative expression. While HMGA1 expression decreased, HMGA2 was progressively upregulated over the time course of hiPSC differentiation towards otic sensory fate. Indeed, we previously reported the onset of HMGA2 expression at E14.5 in initial HCs and supporting cells of sensory epithelia of the embryonic mouse inner ear [48]. Our in vitro results suggest a role for *SOX9*, *SOX11* and *HMGA2* to acquire early otic neurosensory identity.

### 3.4. Expression of HL Genes during hiPSC Differentiation and in Fetal Human Inner Ear

Heterogeneous hearing loss (HL) is evident from mouse screens with multiple new genes underlying both mouse and human auditory dysfunction being uncovered [49]. Our initial studies on human inner ear development were limited to GW 8–12 [50] although hearing function starts at GW19 and balance at GW17. We utilized human inner ear specimens aged GW12 and older from the tissue-bank Human Developmental Biology Resource (HDBR) to address this gap in developmental information. With hiPSCs serving as a stem cell source to generate both neurosensory and associated cellular populations [51], we used it as an in vitro inner ear developmental model to identify genes critical for human otic neurosensory differentiation. Our HTS assay identified constituents of ECM and transcription factors involved in morphogenesis, which were localized, for some of them, in the in vivo fetal human inner ear. Because of composition variability and specificity of ECM in cells and basement membranes, any alteration in component matrix of the cochlear structures can lead to HL. Additionally, ECM influence the fate determination of inner ear progenitors via the RhoA-YAP-β-Catenin signaling. This pathway senses and transmits mechanical cues from ECM, and any deleterious change often results in syndromic HL [30]. ECM-encoding genes (COL2A1 and COL9A2) we identified in HTS and qPCR analyzes during hiPSC differentiation were also previously reported in rodents and human inner ear [32,33,34]. The immunostaining revealed a spatio-temporal expression of ECM (COL2A1, COL9A2 and ITGAV) in fetal human inner ear. Their expression just before the initiation of both hearing and balance suggests a possible role for these markers in inner ear morphogenesis. A loss-of-function mutation in *COL2A1* and *COL9A2* identified as a causative locus in autosomal recessive Stickler syndrome [52] characterized by orofacial and auditory abnormalities. The expression of collagen proteins we observed is indicative of continued procollagen biosynthesis during human fetal stages examined. Additionally, late expression of ITGAV (i.e., GW17) in cochlear HCs suggests an integral role for this integrin subtype during hearing onset. Of interest, immunolocalization of HMGA2 in supporting cells and DACH1 in HCs of the organ of Corti by GW16, suggest their potent roles in sensory differentiation during human inner ear morphogenesis. In addition, HTS/qPCR analysis, revealed an upregulation of *CHD7* at day 6 and day 13 cultures. Mutations in the *CHD7* cause CHARGE syndrome, that affect among other structures, the development of the ear. CHD7 expression in the fetal inner ear could be a sign of maturation and neurogenesis occurring with onset of vestibular and cochlear functions during development. Furthermore, other genes identified by HTS strategy during hiPSC differentiation are likely to have human equivalents that cause HL disorders when mutated. These gene candidates are not necessarily expressed in the HCs or sensory neurons but may lead to auditory and vestibular abnormalities.

## 4. Materials and Methods

### 4.1. Cell Line and Culture Conditions

The hiPSC line (ChiPSC-4) used in this study was provided by Cellectis (formerly Cellartis, now Takara Bio Europe AB, Göteborg, Sweden). The ChiPSC-4 cell line was derived from fibroblasts of healthy human donors and reprogrammed by using polycistronic retrovirus, based on the transduction of Oct3/4, Sox2, Klf4, and c-Myc transcription factors [53]. The ChiPSC-4 hiPSCs were maintained using feeder-free conditions in Cellartis DEF-CSTM 500 Xeno-free GMP grade basal medium (Takara Bio Europe AB) at 37 °C in a humidified atmosphere of 5% CO_2_ in the air with medium changes every day. The hiPSCs were plated at a density of 40,000 cells/cm^2^ onto dishes coated with a DEF-CS™ COAT-1 matrix (Takara Bio Europe AB), diluted at 1:20 in D-PBS (+/+) (Life Technologies, Carlsbad, CA, USA) as previously reported [4]. Cells were expanded in DEF-CS™ 500 basal medium, daily supplemented with DEF-CSTM GF-1 (1:333), GF-2 (1:1000) and GF-3 (1:1000) additives (Takara Bio Europe AB). When the cells were confluent at 80–90% (about 5–7 days), they were passaged using TrypLE Select (Life Technologies). The cells proliferated, and in less than a week, we could proceed to the second passage, seeding the cells on a larger surface, and so on, until having several millions of cells. Pluripotency of reprogrammed cells was confirmed by qPCR and immunostaining.

### 4.2. Otic Placode Induction and Otic Sensory Differentiation

The hiPSCs were dissociated to single cells using TrypLE Select (Life Technologies) and plated on laminin-coated wells (1.5 µg/cm^2^; R&D Systems) at a density of 30,000 cells/cm^2^. For the first day of seeding, cells were cultured in DFNB basal medium (DMEM/F12 with N2/B27) supplemented with fibroblast growth factors, FGF3 (50 ng/mL), FGF10 (50 ng/mL) (R&D Systems) and 10 µM of ROCK inhibitor Y-27632 (StemCell Technologies, Vancouver, BC, Canada) as established in our previous study [4]. From day 2 of differentiation, the medium was replaced with induction medium: DFNB medium with 50 ng/mL FGF3, 50 ng/mL FGF10, 10 µM SB431542 (TGFβ inhibitor, Stemgent) and 100 ng/mL of recombinant human Dickkopf-related protein 1 (rhDKK1) (WNT inhibitor, R&D Systems). The medium was replaced every other day until day 6. Then, the medium was supplemented with 50 ng/mL recombinant human WNT3A (WNT agonist, R&D Systems) instead of SB431542 and rhDkk1. The medium was replaced every 2 days until day 13.

### 4.3. RNA-Seq Data Analysis

Our publicly available RNA-seq data were downloaded from the European Nucleotide Archive (ENA) database (E-MTAB-6679). The RNA extraction procedure, libraries construction, and sequencing are detailed in our previous study [4]. Briefly, the RNAseq libraries (2 × 100 bp; Paired-end) were constructed according to Illumina’s protocol, using 2 µg of total RNA. Sequencing analysis and base calling were performed using the Illumina Pipeline, and sequences were obtained after purity filtering. The studies of the RNAseq experiments were performed with RNAseq data treatment, TopHat (http://tophat.cbcb.umd.edu/, accessed on 1 August 2021) and Cufflinks (http://cufflinks.cbcb.umd.edu/, accessed on 1 August 2021) tools, with adapted and specific parameters (E-MTAB-6679, released on November 2020). To investigate the expression kinetics of candidate genes during differentiation of hiPSCs into otic sensory progenitors, we analyzed the distribution of RNA-seq reads for undifferentiated hiPSCs and differentiated cells at day 6 and day 13 in vitro. We performed Ingenuity Pathway Analysis (IPA) to explore the putative functions of genes and networks that are enriched during hiPSC differentiation. The right-tailed Fisher’s exact test was performed in IPA to calculate a *p*-value determining the probability that each biological function assigned to the data set was due to chance alone. The *p*-values were corrected for multiple testing using the Benjamini-Hochberg method for correcting the FDR, and a *p*-value of <0.05 was set as threshold for statistical significance. Each colored rectangle is a biological function or disease, and the color range indicates its predicted activation state. Blue color indicates a negative z-score and orange color indicate a positive z-score. In this default view, the size of the rectangles is correlated with increasing overlap significance. Regulators with Z-scores greater than 2 or smaller than -2 were considered to be significantly activated or inhibited. Gene Ontology (GO) enrichment analysis was performed using ShinyGO [54] and metascape tools [55]. Volcano plots are generated using START (Shiny Transcriptome Analysis Resource Tool) application [56].

### 4.4. RNA Processing and qRT-PCR

Total RNA was extracted from hiPSCs and differentiated cells using the PureLink^®^ RNA Mini Kit (Life Technologies) according to the manufacturer’s instructions. cDNA was synthesized from 1 µg of RNA per sample, using High-Capacity RNA-to-cDNA™ Kit (Life Technologies). cDNA was diluted 10-fold in DNA suspension buffer (Teknova, Inc, Hollister, CA, USA) and used for the Fluidigm pre-amplification step for Dynamic Array gene expression analysis: cDNA was pre-amplified for 14 cycles with 500 nM DELTA gene pooled primer mix using 2xTaqman PreAmp Master Mix (Invitrogen, Carlsbad, CA, USA), followed by Exo1 treatment (NEB). Five-fold diluted Exo1 treated pre-amplified cDNA was used for loading the 96.96 Dynamic Array chip on the Fluidigm Biomark HD. Primer pairs used for gene expression analysis are listed in the supplemental information (Table S1). The data were analyzed with Real-Time PCR Analysis Software in the BioMark instrument. The Ct values were processed by the automatic threshold for all assays, with derivative baseline correction using BioMark Real-Time PCR Analysis Software 4.1.3 (Fluidigm, South San Francisco, CA, USA).

### 4.5. Ethics Approval of Fetal Specimens

Specimens aged between GW11 and GW12 used in this study were obtained immediately after legal abortion procedures according to the Austrian law (§97StGB of the Austrian Criminal Law as promulgated on 13 November 1998, Federal Law Gazette I). Cadaver donations to the Division of Clinical and Functional Anatomy of the Medical University of Innsbruck for scientific and educational purposes occur only with the informed consent of the donor collected before death. The donors declare during their lifetime that their dead bodies are to be consigned to the anatomical institute for research purposes and the education and advanced training of medical doctors. All embryological body and tissue donations (between the gestational ages 8 to 12) are also released to the anatomical institute by the legally entitled person (mother) accompanied by written consent. Research using these specimens approved by the Ethics Commission of the University Clinic for Gynecology and Obstetrics at the Medical University of Innsbruck, ethical approval number AN2014-0095 335/4.11. In Austria, research projects using these anatomical tissue samples do not need any further approval by an ethics committee of the University. Additional specimens were sourced (between the gestational weeks 8 to 18) from the Human Development Biology Resource (HDBR) branches at UCL London and Newcastle. The human embryonic and fetal material was provided by the Joint MRC/Wellcome Trust (grant# MR/R006237/1) Human Developmental Biology Resource (http://hdbr.org, accessed on 1 August 2021). Abortions were done at Newcastle approved by the North East-Newcastle & North Tyneside 1 Research Ethics committee (ethical approval number 18/NE/0290) and at UCL approved by the Fulham Research Ethics Committee (ethical approval reference no 18/LO/022). Specimens certified by embryologists exhibit no macroscopic malformations, and their embryological ages were differentiated by quantifying features like crown-rump length, external and internal morphology, and the estimated gynecological age. For usage of specimens belonging to the Institute of Pathology of the Medical University of Innsbruck, an ethical commission vote was obtained before commencing this research. This study was approved by the Ethical Commission of the Medical University of Innsbruck (Studienzahl UN2817 Sitzungsnummer 249/4.5 Original Title of the research proposal: *Immunhistochemische Untersuchung von Felsenbeinen mit dazugehöriger Hörbahn*). In Austria, there is no requirement for the consent of the parents or relatives for a clinical autopsy performed in a medical institution § 25 KAKuG Leichenöffnung (Obduktion) (Krankenanstalten- und Kuranstaltengesetz). [(1) The corpses of deceased patients in public hospitals are to be autopsied if the autopsy has been ordered by sanitary or criminal prosecutors, or if it is necessary for public or scientific interests, in the situation of diagnostic uncertainty of the case or after surgical intervention. (2) If none of the cases mentioned in paragraph 1 is present and the deceased has not consented to an autopsy during his lifetime, an autopsy may only be carried out with the consent of the next of kin. (3) A copy of the medical history shall be recorded for each autopsy and kept in accordance with § 10, 1 Z. 3) In the past, § 25 KAKuG was labeled as § 25 KAG (Krankenanstaltengesetz)]. However, in specific cases, a limited autopsy or a postmortem biopsy of one or two organs with a closed body is performed in lieu of a complete autopsy. In Austria, this autopsy procedure allows institutions to keep the autopsy rate to a higher level than other Western countries, permitting high standards of quality assurance and quality controls in most healthcare institutions.

### 4.6. Tissue Preparation, Histology and Immunohistochemistry on Paraffin Sections

Following death, temporal bone specimens were excised immediately and fixed via immersion in a solution of 4% paraformaldehyde (PFA) in phosphate-buffered saline (PBS, 0.1M) overnight. Following rinses with PBS, were dehydrated and embedded in paraffin using a histological infiltration processor (Miles Scientific Inc., Naperville, IL, USA). Using an HM 355S microtome (Microm, Walldorf, Germany), embedded specimens were then serially sectioned at 4 µm thickness and mounted onto Superfrost slides (Menzel, Braunschweig, Germany). Every tenth section was stained with hematoxylin/eosin (Shandon Varistain 24-4, Histocom Vienna, Austria). This enabled orientation and identification of the anatomical landmarks. Chromogenic immunohistochemistry was performed using a Ventana Roche Discovery XT Immunostainer (Mannheim, Germany), applying a DAB-MAP discovery research standard procedure. When required, antigen retrieval was performed by epitope unmasking via a heat induction method performed while the sections were immersed in EDTA buffer (Cell Conditioning Solution CC1, Ventana 950-124). The sections were incubated with the appropriate primary antibodies at 37 °C for 1 h and then with the Discovery Universal Secondary Antibody, Ventana 760-4250 for 30 min. Primary antibodies used in this study are listed in in the supplemental information (Table S2). Antibody detection was then revealed employing the DAB-MAP Detection Kit (Ventana 760-124) using a combinatorial approach involving the diaminobenzidine (DAB) development method with copper enhancement followed by counterstaining with hematoxylin (Ventana 760-2021) for 4 min. The stained sections were dehydrated using an upgraded alcohol series, clarified with xylene and mounted permanently with Entellan (Merck, Darmstadt, Germany). Positive controls (i.e., brain and pancreas) are supplemented to each experiment. Negative controls are acquired by alternating the primary antibodies with reaction buffer or substituting them with isotype-matching immunoglobulins. All sections were digitally examined using a Zeiss AxioVision 4.1 software coupled to an AxioPlan2 microscope (Zeiss, Jena, Germany). The immunostained sections were analyzed at x20 magnification utilizing a TissueFaxs^®^ Plus System coupled onto a Zeiss Axio Imager Z2 Microscope (Jena, Germany). Analyzed areas were then acquired using the TissueFaxs^®^ (TissueGnostics, Vienna, Austria). Fluorescence immunohistochemistry was performed using a Ventana Roche Discovery XT Immunostainer (Mannheim, Germany). The sections were incubated with the primary and secondary antibodies for 1 h. The secondary antibodies used were the donkey anti-rabbit Alexa Fluor488 (ThermoFischer Scientific, Vienna, Austria), donkey anti-rabbit Alexa Fluor594 (ThermoFischer Scientific) and donkey anti-mouse Alexa Fluor488 (ThermoFischer Scientific). Immunostained sections were digitized at 40× and 63× magnifications using a TissueFAXS Plus System coupled to Zeiss Axio Imager Z2 microscope (Jena, Germany). Image acquisition was performed using the Tissue FAXS software (TissueGnostics, Vienna, Austria).

## 5. Conclusions

The present study reveals for the first time important upregulation of cell-to-substrate adhesion molecules, including ECM and ITG genes that coincides with the expression of otic neurosensory developmental genes during hiPSC differentiation. The ubiquity of their expression and the multiple modes by which they operate suggest a fundamental role of ECM and ITG molecules in promoting otic neurosensory fate during hiPSC differentiation in vitro and likely in vivo in the human fetal inner ear. An improved understanding of ITG-mediated cell-ECM interaction and ITG signaling may help to increase the specificity and efficiency of directing hiPSC into neurosensory lineage for human inner ear development and regeneration studies.

## Supplementary Materials

The following are available online at https://www.mdpi.com/article/10.3390/ijms221910849/s1.
