# Supplementary material for: Transcriptome-Wide Analysis Reveals a Role for Extracellular Matrix and Integrin Receptor Genes in Otic Neurosensory Differentiation from Human iPSCs
Source: Int J Mol Sci. 2021 Oct 7;22(19):10849. doi: 10.3390/ijms221910849 (PMC8509699; doi:10.3390/ijms221910849)
Supplement: Supplementary file 1 [file ijms-22-10849-s001.zip › Suppl Mat.pdf]

Figure S1. Functional annotation clustering by GO of differentiated hiPSCs at day-6 *in vitro* relative to undiffrentiated hiPSCs. (A) GO analysis identifies enrichment of top cellular component during hiPSC differentiation into otic sensory cells. The ECM and collagens are among the top clusterd cellular components. The size of the dots represents the gene numbers annotated in this cellular component term. (B) Network visualization of enriched cellular components performed by GO analysis revealed the ECM and collagen-containing ECM are the highly enriched cellular components in the gene signature of otic sensory cells at day-6 compared to undifferentiated hiPSCs.

Figure S2. Analysis of significantly represented GO terms using IPA. The heat map colors correspond to the relative expression of genes represented in the subgroups of the major annotations terms. Most 'cellular and tissue development', 'cellular movement', and 'cell-to-cell signaling' genes were upregulated in day-13 *in vitro* compared to those of undifferentiated hiPSCs at day-0. Orange color indicates upregulated genes; blue color indicates downregulated genes. GO: gene ontology; IPA: ingenuity pathway analysis.

Figure S3. Functional network analysis related to upregulated genes in day-6 and day-13 cell cultures. Characterization of *HMGA2*, *DACH1* and *BMP* and their interactions with ECM during hiPSC differentiation of hiPSCs to human otic sensory cells. Genes shown in pink are up-regulated and in green are downregulated genes in day-6 and day-13 *in vitro*.

Figure S4. Functional network analysis related to gene markers upregulated during otic differentiation from hiPSCs. Significant network and genes assembled around Collagen, Cadherin, RA (*RDH10, CYP26B*,..) and SOX genes progressivelly upregulated during hiPSC differentiation. Nodes shaded in pink represent genes that are upregulated at day-6 and day-13 *in vitro*, and green nodes are genes that are downregulated at day-6 and day-13 *in vitro*. These networks assembled by up- and down-regulated genes. The intensity of the node color indicates the degree of gene upregulation or downregulation. Edges (lines) and nodes are annotated with labels that illustrate the nature of the relationship between genes and their functions. A solid line represents a direct interaction and a dotted line an indirect interaction.

Figure S5. Functional network analysis related to markers upregulated during otic differentiation. Significant network and genes assembled around collagen (*COL2A1*, *COL3A1*,..) and otic placodal (*OTX1*, *SIX1*,..) gene markers that are gradually upregulated during the time course of hiPSC differentiation. The intensity of the node color indicates the degree of gene up or down-regulation. Edges (lines) and nodes are annotated with labels that illustrate the nature of the relationship between genes and their functions. A solid line represents a direct interaction and a dotted line an indirect interaction.
