# Supplementary material for: Transcriptome-Wide Analysis Reveals a Role for Extracellular Matrix and Integrin Receptor Genes in Otic Neurosensory Differentiation from Human iPSCs
Source: Int J Mol Sci. 2021 Oct 7;22(19):10849. doi: 10.3390/ijms221910849 (PMC8509699; doi:10.3390/ijms221910849)
Supplement: Supplementary file 1 [file ijms-22-10849-s001.zip › Table S1.pdf]

Supporting Table S1 : Primers for RT-qPCR validation

GENE **(HUGO ID.)**	**INFO**	**ANNOTATION**	**TAQMAN ASSAY ID**
**HMGA2**	Target 1	chromodomain helicase DNA binding protein 7 (HGNC:20626)	Hs04397751_m1
**DACH1**	Target 2	dachshund family transcription factor 1 (HGNC:2663)	Hs00974297_m1
**CHD7**	Target 3	high mobility group AT-hook 2 (HGNC:5009)	Hs00215010_m1
**BMP7**	Target 4	bone morphogenetic protein 7 (HGNC:1074)	Hs00233476_m1
**SOX9**	Target 5	SRY-box transcription factor 9 (HGNC:11204)	Hs00165814_m1
**SOX2**	Target 6	SRY-box transcription factor 2 (HGNC:11195)	Hs01053049_s1
**SOX11**	Target 7	SRY-box transcription factor 11 (HGNC:11191)	Hs00846583_s1
**CYP26B1**	Target 8	cytochrome P450 family 26 subfamily B member 1	Hs01011223_m1
**RDH10**	Target 9	retinol dehydrogenase 10 (HGNC:19975)	Hs00416907_m1
**RAI1**	Target 10	retinoic acid induced 1 (HGNC:9834)	Hs01554690_m1
**ITGAV**	Target 11	integrin subunit alpha V (HGNC:6150)	Hs00233808_m1
**ITGB4**	Target 12	integrin subunit beta 4 (HGNC:6158)	Hs00236216_m1
**NANOG**	Reference D0	Nanog homeobox (HGNC: 20857)	Hs02387400_g1
**DLX5**	Reference D6	Distal-less homeobox 5 (HGNC: 2918)	Hs01573641_mH
**WNT7B**	Reference D13	Wingless-type MMTV integration site family, member 7B	Hs00536497_m1
**GAPDH**	Internal control	Glyceraldehyde-3-phosphate dehydrogenase (HGNC: 4141)	Hs02786624_g1

GENE (HUGO ID.)	INFO	ANNOTATION	TAQMAN ASSAY ID
**LUM**	Target 1	Lumican	Hs00929860_m1
**DCN**	Target 2	Decorin	Hs00754870_s1
**DNMT3B**	Target 3	DNA (cytosine-5-)-methyltransferase 3 beta	Hs00171876_m1
**COL1A2**	Target 4	Collagen, type I, alpha 2	Hs01028956_m1
**COL2A1**	Target 5	Collagen, type II, alpha 1	Hs00264051_m1
**COL12A1**	Target 6	Collagen, type XII, alpha 1	Hs00189184_m1
**COL3A1**	Target 7	Collagen, type III, alpha 1	Hs00943809_m1
**COL5A1**	Target 8	Collagen, type V, alpha 1	Hs00609133_m1
**KRT8**	Target 9	Keratin 8	Hs01595539_g1
**FN1**	Target 10	Fibronectin 1	Hs01549976_m1
**VIM**	Target 11	Vimentin	Hs00958111_m1
**NANOG**	reference DO	Nanog homeobox	Hs02387400_g1
**DLX5**	reference D6	Distal-less homeobox 5	Hs01573641_mH
**WNT7B**	reference D13	Wingless-type MMTV integration site family, member 7B	Hs00536497_m1
**GAPDH**	Internal control	Glyceraldehyde-3-phosphate dehydrogenase	Hs02786624_g1
