# Supplementary material for: Transcriptome-Wide Analysis Reveals a Role for Extracellular Matrix and Integrin Receptor Genes in Otic Neurosensory Differentiation from Human iPSCs
Source: Int J Mol Sci. 2021 Oct 7;22(19):10849. doi: 10.3390/ijms221910849 (PMC8509699; doi:10.3390/ijms221910849)
Supplement: Supplementary file 1 [file ijms-22-10849-s001.zip › Table S2.pdf]

Table S2. The hosts, dilutions, and sources of primary antibodies used in the study

Antibody clone	Localization pattern	Host description	Background	Antibody Dilution Pre-treatment	Supplier Cat. No:
DACH1	Nuclear	Rabbit polyclonal	Liu et al, 2015<br>PMID:25940701	1:250<br>CC1 standard	Proteintech<br>10914-1-AP
BMP7	Nuclear	Mouse monoclonal	Hsu et al, 2008<br>PMID:18560367	1:500<br>CC1 standard	R&D System<br>MAB3541
HMGA2	Nuclear	Rabbit polyclonal	Li et al, 2020<br>PMID:31966084	1:4000<br>CC1 standard	Proteintech<br>20795-1-AP
CHD7	Nuclear	Rabbit polyclonal	Janssen et al,2012<br>PMID:22461308	1:100<br>CC1 standard	Abcam 117522
COL2A1	Cytoplasmic	Rabbit<br>polyclonal	Crombrugghe et al, 2000<br>PMID:10980415	2,5µl/400µl<br>CC1 standard	Sigma<br>HPA055753
COL9A2	Cytoplasmic	Rabbit<br>polyclonal	Hartman et al,2015<br>PMID:25852475	2,5µl/400µl<br>CC1 standard	Sigma<br>HPA056316
ITGAV	Cytoplasmic	Rabbit	Morandi et al,2016	2,5µl/400µl	Sigma

		polyclonal	PMID:27363302	CC1 standard	HPA004856
Neuron specific beta 3 tubulin (TUBB3)	Cytoplasmic	Mouse monoclonal	Sengle et al,2008<br>PMID:18621057	1:160<br>CC1 standard	R&D System MAB1195
