# Supplementary figures and images for: Transcriptome-Wide Analysis Reveals a Role for Extracellular Matrix and Integrin Receptor Genes in Otic Neurosensory Differentiation from Human iPSCs
Source: Int J Mol Sci. 2021 Oct 7;22(19):10849. doi: 10.3390/ijms221910849 (PMC8509699; doi:10.3390/ijms221910849)

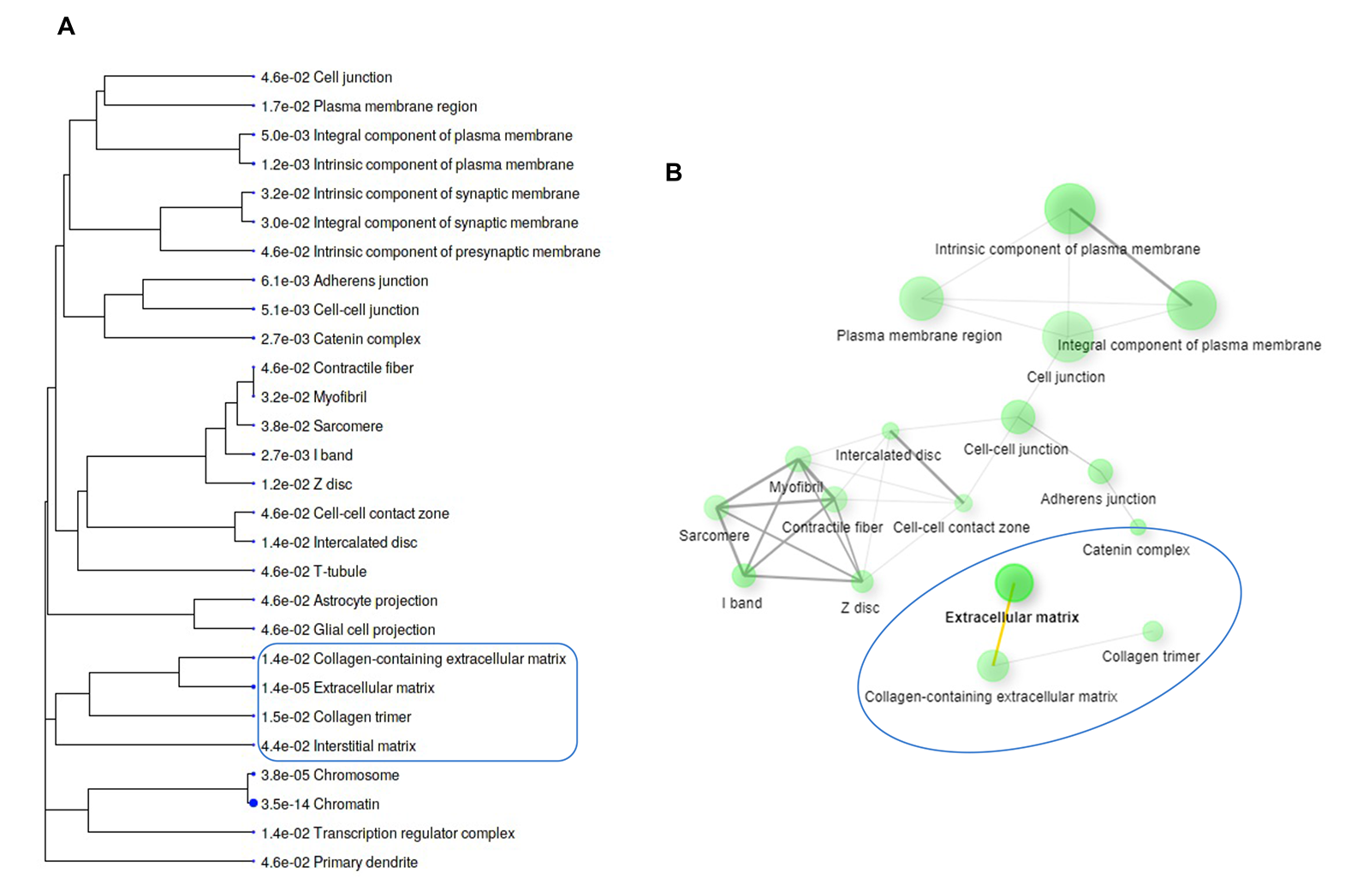

Supplement: Supplementary file 1 [file ijms-22-10849-s001.zip › Figure S1.tif]

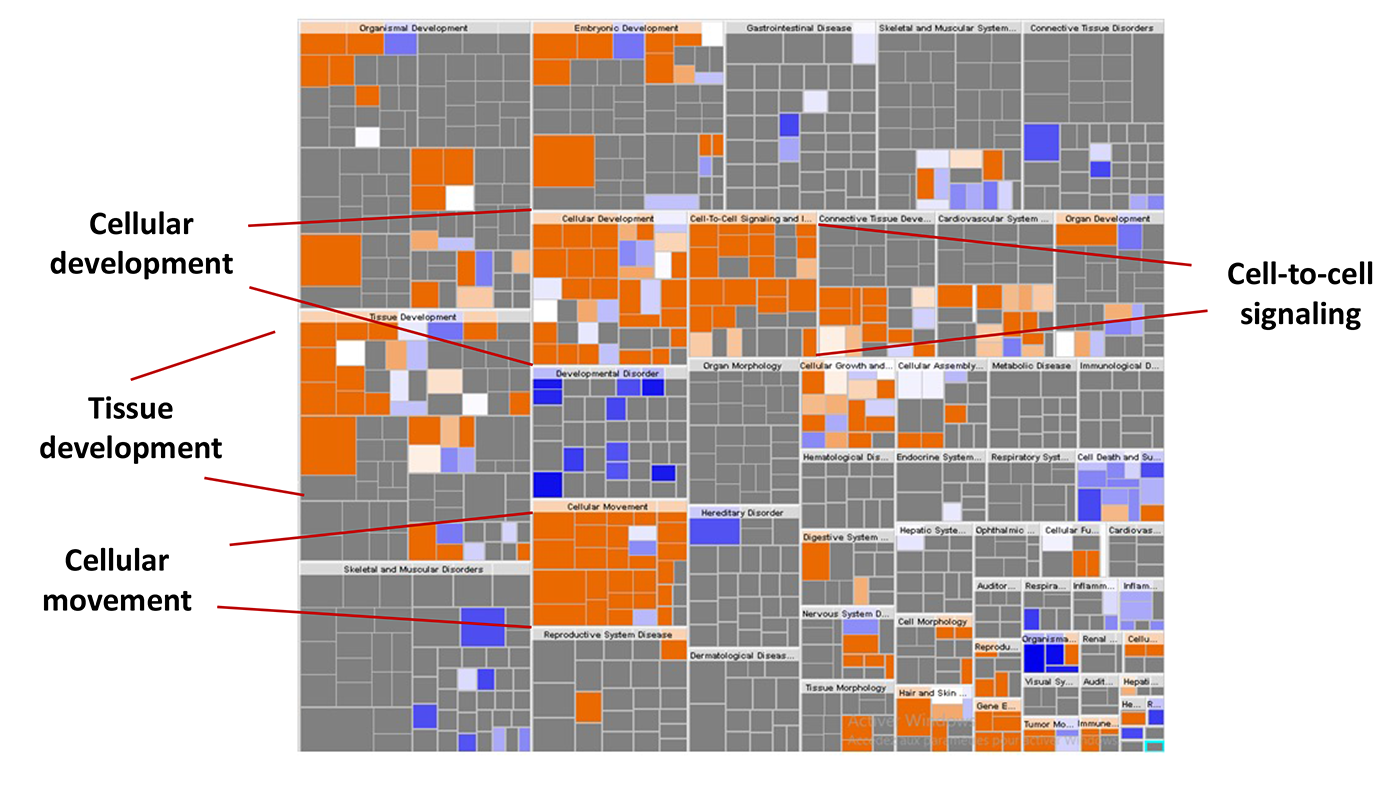

Supplement: Supplementary file 1 [file ijms-22-10849-s001.zip › Figure S2.tif]

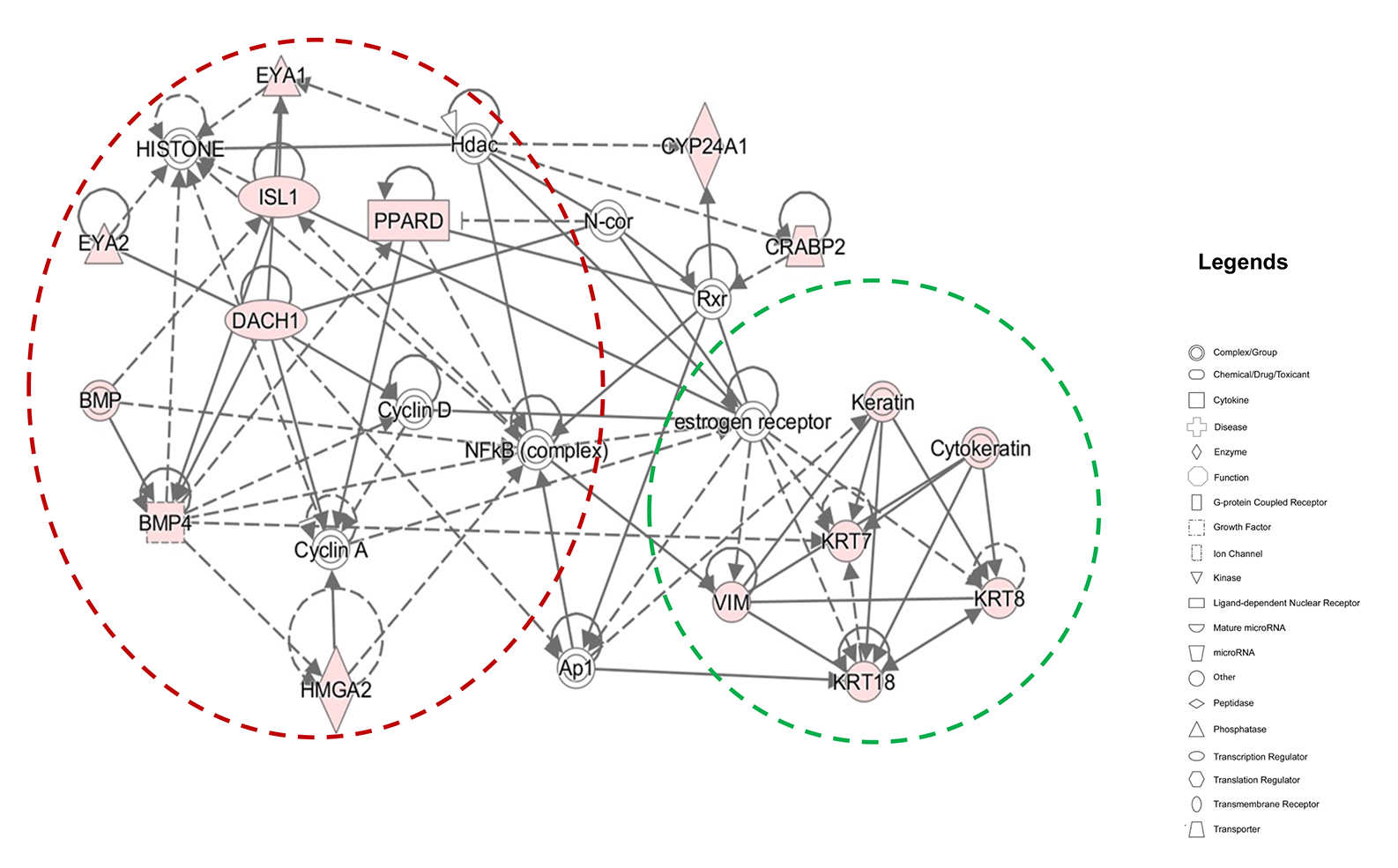

Supplement: Supplementary file 1 [file ijms-22-10849-s001.zip › Figure S3.tif]

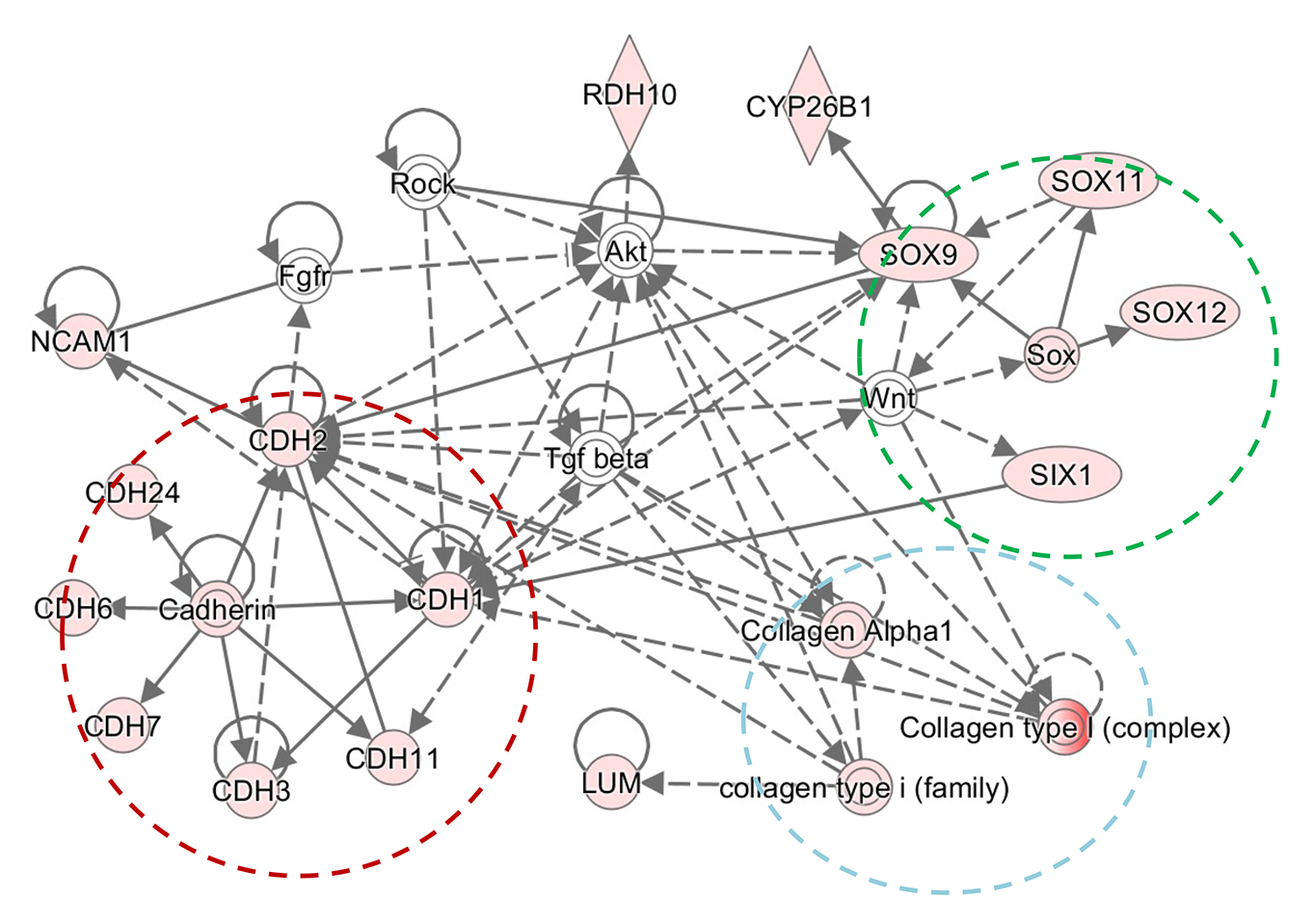

Supplement: Supplementary file 1 [file ijms-22-10849-s001.zip › Figure S4.tif]

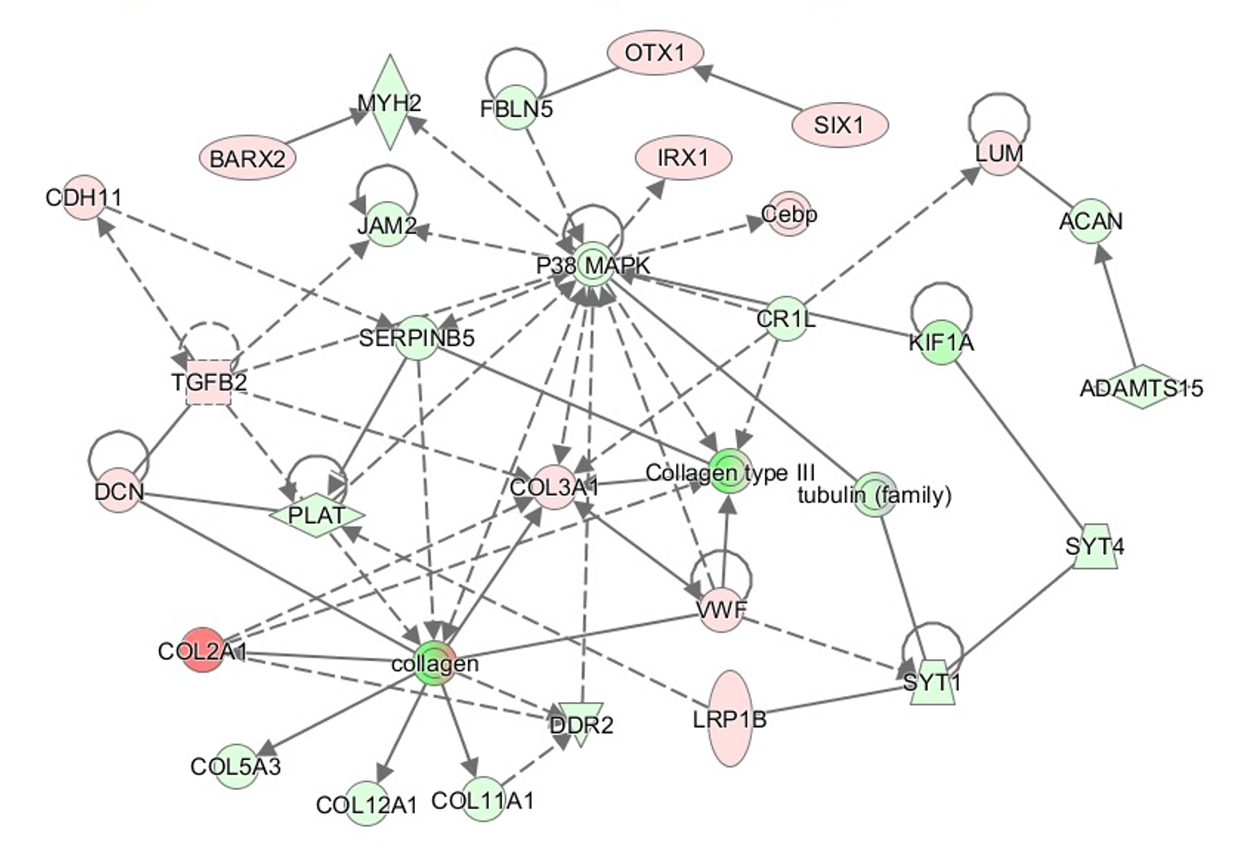

Supplement: Supplementary file 1 [file ijms-22-10849-s001.zip › Figure S5.tif]
